# An Uncertain Cardiac Mass: A Case of Whipple’s Disease

**DOI:** 10.7759/cureus.105333

**Published:** 2026-03-16

**Authors:** Jessica Fidalgo, Carina Santos, Sonia Coelho, Catarina Quinaz

**Affiliations:** 1 Internal Medicine, Unidade Local de Saúde (ULS) Guarda, Guarda, PRT; 2 Infectious Disease, Unidade Local de Saúde (ULS) Guarda, Guarda, PRT

**Keywords:** multidisciplinary decision-making, subacute endocarditis, surgical valve replacement, valvular regurgitation, whipple's disease

## Abstract

Whipple’s disease is a rare multisystem infectious disorder with a heterogeneous clinical presentation that frequently leads to delayed diagnosis. Relapses may occur even years after apparent remission. We report a 54-year-old man with a previous diagnosis of Whipple’s disease who presented with fatigue, weight loss, anemia, and recurrent gastrointestinal symptoms. Echocardiography showed a persistent aortic valve mass with progressive regurgitation in the setting of repeatedly negative blood cultures and minimal systemic inflammatory response. Upper gastrointestinal endoscopy with duodenal biopsies revealed periodic acid-Schiff-positive macrophages, and real-time polymerase chain reaction confirmed *Tropheryma (T.) whipplei* in duodenal tissue. The patient received intravenous ceftriaxone followed by oral doxycycline plus hydroxychloroquine and underwent mechanical aortic valve replacement; polymerase chain reaction of the excised valve was also positive for *T. whipplei*. This case emphasizes the need to consider *T. whipplei* in indolent, culture-negative endocarditis and highlights the role of molecular testing and multidisciplinary management.

## Introduction

Whipple’s disease is a rare, chronic multisystem infection caused by the Gram-positive Actinomycete *Tropheryma (T.) whipplei*. The disease predominantly affects middle-aged men and classically presents with a constellation of systemic manifestations, including weight loss, chronic diarrhea, abdominal pain, arthralgia, and malabsorption [1]. Histologically, it is characterized by the accumulation of periodic acid-Schiff (PAS)-positive macrophages within affected tissues, most commonly the small intestinal mucosa. The pathogenesis is thought to involve impaired cellular immune responses that allow persistence and systemic dissemination of the organism [1,2].

Although gastrointestinal manifestations are the most recognized features, Whipple’s disease can involve multiple organ systems, including the central nervous system, joints, lymphatic system, and cardiovascular system [2]. Cardiac involvement may occur in the form of endocarditis, myocarditis, or pericarditis, with endocarditis representing the most frequently described cardiac manifestation. Notably, *T. whipplei* has emerged as an important cause of blood culture-negative infective endocarditis, often presenting with an indolent clinical course and minimal systemic inflammatory response [3]. *T. whipplei *accounts for up to 6% of cases in some molecular diagnostic series [4]. Patients may lack classic signs of infection, which can delay diagnosis and lead to misinterpretation of valvular lesions as thrombi or benign cardiac masses.

The diagnosis of Whipple’s disease relies on a combination of histopathological findings and molecular detection of *T. whipplei*. Duodenal biopsies demonstrating PAS-positive macrophages remain a cornerstone of diagnosis, while polymerase chain reaction (PCR) assays targeting *T. whipplei* have shown high diagnostic sensitivity and specificity and have significantly improved diagnostic accuracy, particularly in cases with extraintestinal involvement or atypical presentations [1,3]. In patients with suspected infective endocarditis and persistently negative blood cultures, molecular testing of tissue specimens, including excised cardiac valves, may be crucial for establishing the diagnosis.

We report the case of a 54-year-old man with a prior history of Whipple’s disease who presented with fatigue, weight loss, and anemia. Transthoracic echocardiography revealed a mobile mass attached to the aortic valve compatible with vegetation, associated with moderate-to-severe aortic regurgitation (grade III/IV). The left ventricle was mildly dilated (LV end-diastolic diameter 60 mm) with preserved systolic function (ejection fraction 78%), and no significant transvalvular gradient was observed. Duodenal biopsy demonstrated periodic acid-Schiff-positive macrophages, and polymerase chain reaction (PCR) testing confirmed *T. whipplei *infection in both duodenal tissue and the excised aortic valve. The patient was treated with intravenous ceftriaxone (2 g/day), followed by doxycycline (100 mg twice daily) and hydroxychloroquine (200 mg three times daily). He subsequently underwent mechanical aortic valve replacement through a median sternotomy, with an operative time of 128 minutes and an uneventful postoperative course, requiring a three-day stay in the intensive care unit.

## Case presentation

A 54-year-old man with no relevant prior medical history was first hospitalized in December 2018 at another acute care hospital for anasarca and diagnosed with minimal change glomerulonephritis. Transthoracic echocardiography performed during this admission revealed a mobile mass measuring 10 × 6 mm on the right coronary cusp of the aortic valve. After a cardiology consultation, the lesion was interpreted as an intracardiac thrombus, and anticoagulation with warfarin was initiated. 

**Figure 1:**
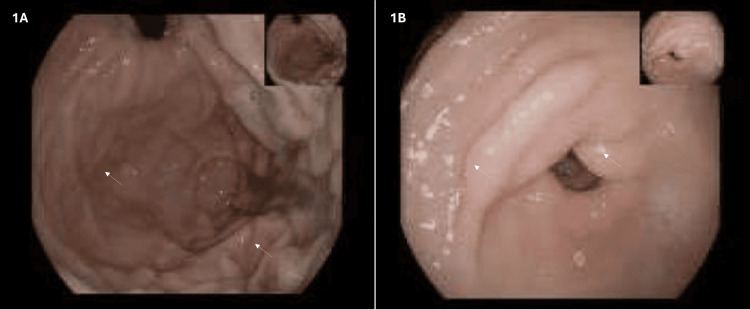
Upper gastrointestinal endoscopy (A) Endoscopic view of the duodenal mucosa showing irregular mucosal folds and edema (arrows). (B) Additional endoscopic view demonstrating subtle mucosal abnormalities of the duodenum (arrows).

Later that month, the patient was readmitted with epigastric pain, fatigue, and anemia. Upper gastrointestinal endoscopy showed edematous and friable duodenal mucosa with multiple lymphangiectasias (Figure 1). Duodenal biopsy demonstrated histiocytic cells with periodic acid-Schiff (PAS)-positive cytoplasmic granules, suggestive of Whipple’s disease. Polymerase chain reaction (PCR) testing for *T. whipplei* on duodenal tissue was positive. Representative histopathological images were not available for publication. Treatment with trimethoprim-sulfamethoxazole was initiated in February 2019.

Follow-up echocardiography showed persistence of the aortic valve mass, although reduced in size, with associated severe aortic regurgitation based on echocardiographic assessment. In 2020, after one year of trimethoprim-sulfamethoxazole, endoscopic abnormalities persisted, and therapy was changed to doxycycline.

Despite one year of doxycycline, duodenal biopsies in 2021 remained compatible with Whipple’s disease, and the patient was referred for infectious diseases but was subsequently lost to follow-up.

In August 2025, the patient presented in our hospital with a two-month history of abdominal pain with daily loose and greasy stools, intermittent abdominal pain, and a 30 kg weight loss, and was found to be anemic. Given the previous history, the entire work-up was restarted, including baseline endoscopic assessment with PCR testing for *T. whipplei*, exclusion of central nervous system infection, repeated endocardial assessment of the mass previously interpreted as a thrombus, and planning of hospital admission for further investigation and rescue treatment. This admission was brought forward due to clinical deterioration and the identification of an image compatible with vegetation in the echocardiogram performed in the outpatient setting, reporting moderate-to-severe aortic regurgitation.

On examination, he was afebrile (37.7 °C), with a grade III/VI holosystolic murmur. Laboratory testing revealed leukocytosis, normocytic normochromic anemia, hypokalemia, and mildly elevated inflammatory markers (Table 1). Blood cultures were negative.

**Table 1:** Laboratory findings at admission

Parameter	Patient value	Reference range
Leukocytes	10.77 × 10⁹/L	4.0–10.0 × 10⁹/L
Hemoglobin	10.4 g/dL	13.0–17.0 g/dL
Potassium	3.2 mmol/L	3.5–5.0 mmol/L
C-reactive protein	1.45 mg/dL	<0.5 mg/dL

**Figure 2:**
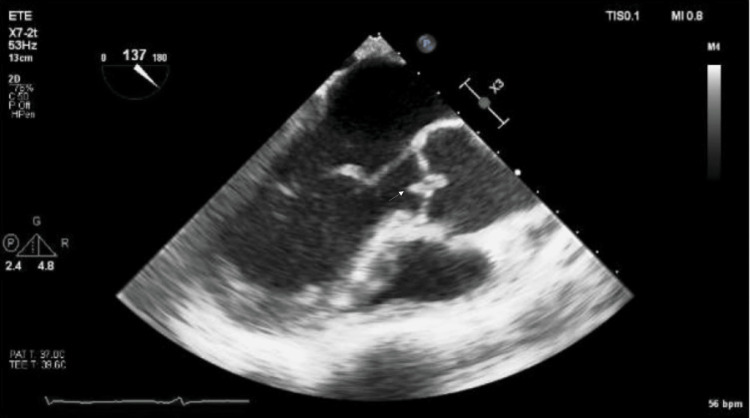
Transesophageal echocardiography Transesophageal echocardiography demonstrating a rounded lesion measuring 0.26 cm² attached to the right coronary cusp of the aortic valve (arrow), associated with moderate-to-severe aortic regurgitation.

Transesophageal echocardiography demonstrated a rounded lesion measuring 0.26 cm² attached to the right coronary cusp of the aortic valve, with moderate-to-severe aortic regurgitation (Figure 2). Given the chronicity of the lesion, absence of sustained fever, negative blood cultures, and low inflammatory markers, the differential diagnosis included thrombus versus vegetation. After a multidisciplinary discussion, Whipple’s disease-associated endocarditis was considered the most likely diagnosis.

Lumbar puncture with PCR testing for *T. whipplei* in cerebrospinal fluid was negative. Upper gastrointestinal endoscopy confirmed relapse of Whipple’s disease, with duodenal biopsies showing foamy PAS-positive macrophages and positive real-time PCR for *T. whipplei*. The patient received 14 days of intravenous ceftriaxone due to the inadequate previous regimens, followed by oral doxycycline (100 mg twice daily) combined with hydroxychloroquine (200 mg three times daily). During hospitalization, the patient completed 54 days of doxycycline therapy, which was continued after discharge as long-term treatment for *T. whipplei* endocarditis.

Ophthalmologic evaluation before hydroxychloroquine initiation was unremarkable, and serial QT-interval monitoring showed no abnormalities.

Given severe aortic regurgitation, the patient underwent mechanical aortic valve replacement after preoperative evaluation. PCR testing of the excised valve was positive for *T. whipplei*. The patient had a favorable postoperative course, with normalization of inflammatory markers, and was discharged on warfarin with therapeutic anticoagulation and continued doxycycline (100 mg twice daily) and hydroxychloroquine (200 mg three times daily), planned for long-term therapy for at least 18 months, with follow-up in internal medicine and infectious diseases clinics.

The clinical course from the initial diagnosis of Whipple’s disease to the development of infective endocarditis is summarized in Table 2.

**Table 2:** Timeline of clinical course Timeline of clinical course from the initial diagnosis of Whipple’s disease to the development of *Tropheryma whipplei *endocarditis and surgical valve replacement.

Date	Clinical Event
Dec 2018	Hospitalization for anasarca; minimal change glomerulonephritis diagnosed. Transthoracic echocardiography revealed a mobile aortic valve mass (10 × 6 mm), initially interpreted as thrombus.
Dec 2018	Upper GI endoscopy with duodenal biopsy confirmed Whipple’s disease (PAS-positive macrophages; PCR positive for *T. whipplei*).
Feb 2019	Treatment initiated with trimethoprim-sulfamethoxazole.
2020	Persistent endoscopic abnormalities; therapy changed to doxycycline.
2021	Duodenal biopsies remained compatible with Whipple’s disease; the patient was lost to follow-up.
Aug 2025	Readmission with abdominal pain, steatorrhea, anemia, and 30 kg weight loss.
Sept 2025	Echocardiography revealed aortic valve vegetation with moderate-to-severe regurgitation.
Sept 2025	Duodenal biopsy and PCR confirmed relapse of Whipple’s disease.
Oct 2025	Treated with intravenous ceftriaxone, followed by doxycycline + hydroxychloroquine.
Oct 2025	Mechanical aortic valve replacement performed. PCR of the excised valve was positive for *T. whipplei*.

## Discussion

Whipple’s disease is a rare, chronic, multisystem infectious disorder whose diagnosis is frequently delayed because of its heterogeneous and nonspecific clinical presentation with several extra-intestinal manifestations that may manifest in the absence of gastrointestinal symptoms at the time of diagnosis. Although classically associated with malabsorption and arthropathy, *T. whipplei *infection is increasingly recognized as an important cause of indolent, culture-negative endocarditis [2,3,5], often presenting primarily with cardiac manifestations, such as progressive valvular dysfunction or heart failure, rather than systemic inflammatory features [6,7]. In some cases, cardiac or central nervous system involvement may represent the only clinical manifestations, occurring in the absence of gastrointestinal symptoms. In such situations, molecular techniques, such as PCR, performed on tissue samples play a crucial role in identifying the pathogen.

Although gastrointestinal and articular manifestations are the most frequently recognized features of Whipple’s disease, other organ systems may be involved. Renal manifestations are rare but have been described, including cases of glomerulonephritis, which are thought to result from immune-mediated mechanisms or systemic dissemination of *T. whipplei*. Such atypical presentations may contribute to diagnostic delay and obscure the underlying infectious etiology.

Cardiac involvement in Whipple’s disease has been reported in up to 50% of patients with classic disease [2], yet isolated or predominant endocarditis remains underrecognized [3]. Other rare cardiac manifestations have also been described, including constrictive myopericarditis presenting with right-sided heart failure, which in selected cases has been successfully managed with medical therapy alone without the need for pericardiectomy [8]. In such cases, the absence of fever, persistently negative blood cultures, and low inflammatory markers may obscure the infectious nature of valvular lesions and promote diagnostic anchoring toward noninfectious etiologies, including intracardiac thrombus or benign tumors such as fibroelastoma [3,5,6]. Several published cases and series describe a prolonged, indolent course, with vegetations or valvular abnormalities detectable months or years before diagnosis, frequently culminating in severe regurgitation and the need for surgical intervention [6,7,9]. Definitive diagnosis of *T. whipplei* endocarditis relies on molecular detection of the organism, most reliably performed on resected valvular tissue [3]. Polymerase chain reaction testing has emerged as the diagnostic cornerstone, given the uniformly negative blood cultures and the limitations of serologic testing. PCR assays for *T. whipplei *show high diagnostic sensitivity and specificity, particularly when performed on tissue samples [3]. Contemporary guidelines for the management of infective endocarditis emphasize the systematic evaluation of rare causes in blood culture-negative endocarditis, including *T. whipplei*, particularly when echocardiographic abnormalities persist in the setting of minimal systemic inflammation [10].

Relapse and persistent infection represent major challenges in Whipple’s disease and are thought to reflect both intracellular persistence of the organism and host immune dysregulation [2]. Observational data suggest that incomplete eradication, interruptions in therapy, or loss to follow-up are associated with an increased risk of recurrence, underscoring the need for prolonged combination antimicrobial therapy and structured long-term surveillance [2,7]. Surgical valve replacement, when indicated, may play a critical role not only in hemodynamic stabilization but also in achieving microbiologic cure [3,6].

The treatment of Whipple’s disease-associated endocarditis is challenging and remains guided largely by observational data and expert opinion, owing to the rarity of the condition and the absence of randomized clinical trials. Prolonged antimicrobial therapy is required to achieve sustained eradication of *T. whipplei*, reflecting the organism’s intracellular persistence and the risk of relapse.

According to contemporary European Society of Cardiology guidelines, the treatment of *T. whipplei* endocarditis typically includes an initial course of intravenous antibiotics, most commonly ceftriaxone or penicillin G, particularly in cases with cardiac or central nervous system involvement. This is followed by prolonged oral therapy to ensure the eradication of the intracellular organism. Long-term treatment with doxycycline combined with hydroxychloroquine is currently recommended for at least 18 months, although the optimal duration of therapy remains uncertain. Trimethoprim-sulfamethoxazole has also been used historically as maintenance therapy in Whipple’s disease [9].

This case reinforces the importance of maintaining a high index of suspicion for *T. whipplei* infection in patients with culture-negative endocarditis, particularly in those with systemic features such as weight loss, malabsorption, arthralgia, or a history of Whipple’s disease. 

Early recognition, molecular confirmation, prolonged antimicrobial therapy, and coordinated multidisciplinary follow-up are essential to improving outcomes in this potentially fatal yet treatable condition.

In patients with indolent, blood culture-negative endocarditis, particularly when systemic features, such as weight loss, malabsorption, arthralgia, or a prior history of Whipple’s disease are present, *T. whipplei* should remain in the differential diagnosis. Cardiac involvement may manifest as persistent valvular lesions with little or no inflammatory response, which can delay recognition and lead to misclassification as thrombus or benign intracardiac masses. Molecular testing, especially PCR performed on tissue (duodenal biopsies and, when available, excised valve material), is pivotal for confirmation. Management typically requires prolonged targeted antimicrobial therapy and may need surgical valve replacement when significant valvular dysfunction develops, reinforcing the importance of multidisciplinary follow-up.

## Conclusions

Whipple’s disease remains an uncommon but important cause of culture-negative infective endocarditis and may present with nonspecific systemic manifestations, which can delay recognition. This case highlights the diagnostic challenge posed by indolent valvular lesions associated with minimal inflammatory response and persistently negative blood cultures. In such settings, *Tropheryma whipplei *should be considered in the differential diagnosis, particularly in patients with compatible systemic symptoms or a previous history of Whipple’s disease.

Molecular diagnostic techniques, especially PCR performed on tissue samples, such as duodenal biopsies or excised cardiac valves, play a pivotal role in establishing the diagnosis. Because gastrointestinal biopsies may be negative in localized forms of the disease, analysis of cardiac tissue, including PAS staining and PCR, may be essential for confirming *Tropheryma whipplei* infection. Early recognition, appropriate prolonged antimicrobial therapy, and timely surgical management when indicated are essential to improve outcomes and prevent disease progression. A better understanding of the underlying immunopathogenesis, including macrophage dysfunction, impaired Th1 immune responses, and the potential development of immune reconstitution inflammatory syndrome (IRIS), may further improve disease recognition and guide therapeutic strategies.

## References

[1] Marth T, Moos V, Müller C, Biagi F, Schneider T (2016). Tropheryma whipplei infection and Whipple disease. Lancet Infect Dis.

[2] Fenollar F, Puéchal X, Raoult D (2007). Whipple's disease. N Engl J Med.

[3] Herrmann MD, Neumayr A, Essig A (2014). Isolated Whipple's endocarditis: an underestimated diagnosis that requires molecular analysis of surgical material. Ann Thorac Surg.

[4] Geissdörfer W, Moos V, Moter A (2012). High frequency of Tropheryma whipplei in culture-negative endocarditis. J Clin Microbiol.

[5] Knol S, Nijhuis RL, Geeraedts F, Linssen GC (2021). Blood culture-negative endocarditis caused by Tropheryma whipplei: Whipple's endocarditis. Eur J Case Rep Intern Med.

[6] Naso JR, Wong D, Wong DR (2019). Tropheryma whipplei endocarditis presenting as chronic valvular disease: a case report and review of the literature. Hum Pathol Case Rep.

[7] McGee M, Brienesse S, Chong B, Levendel A, Lai K (2019). Tropheryma whipplei endocarditis: case presentation and review of the literature. Open Forum Infect Dis.

[8] Marot J, Pierard S, Bamps L, Yildiz H, Yombi JC (2025). Whipple's disease, a rare cause of constrictive myopericarditis and right-sided heart failure. Cureus.

[9] Sullivan A, Shrestha P, Basnet S, Herb R, Zagorski E (2020). A rare case of Whipple's disease with endocarditis in a patient with dextrocardia. SAGE Open Med Case Rep.

[10] Delgado V, Ajmone Marsan N, de Waha S (2023). 2023 ESC guidelines for the management of endocarditis: developed by the task force on the management of endocarditis of the European Society of Cardiology (ESC) endorsed by the European Association for Cardio-Thoracic Surgery (EACTS) and the European Association of Nuclear Medicine (EANM). Eur Heart J.

